# Identifying Process Differences with ToF-SIMS: An MVA Decomposition Strategy

**DOI:** 10.1021/jasms.4c00327

**Published:** 2024-10-04

**Authors:** Nico Fransaert, Allyson Robert, Bart Cleuren, Jean V. Manca, Dirk Valkenborg

**Affiliations:** † UHasselt, X-LAB, Agoralaan, 3590 Diepenbeek, Belgium; ‡ UHasselt, Theory Lab, Agoralaan, 3590 Diepenbeek, Belgium; ¶ UHasselt, Data Science Institute, Interuniversity Institute for Biostatistics and Statistical Bioinformatics, Center for Statistics, Agoralaan, 3590 Diepenbeek, Belgium

**Keywords:** time-of-flight secondary ion mass spectrometry, mass spectrometry, multivariate analysis, spectral analysis, principal component analysis, partial least-squares discriminant analysis, data analysis, decomposition, feature extraction, discrimination, dye-sensitized solar cells, N719 dye, photodegradation

## Abstract

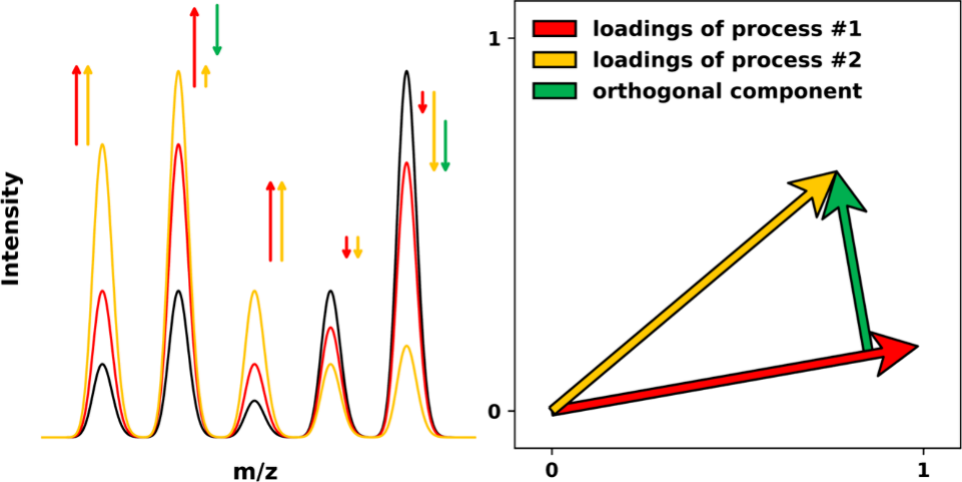

In time-of-flight secondary ion mass spectrometry (ToF-SIMS), multivariate analysis (MVA) methods such as principal component analysis (PCA) are routinely employed to differentiate spectra. However, additional insights can often be gained by comparing processes, where each process is characterized by its own start and end spectra, such as when identical samples undergo slightly different treatments or when slightly different samples receive the same treatment. This study proposes a strategy to compare such processes by decomposing the loading vectors associated with them, which highlights differences in the relative behavior of the peaks. This strategy identifies key information beyond what is captured by the loading vectors or the end spectra alone. While PCA is widely used, partial least-squares discriminant analysis (PLS-DA) serves as a supervised alternative and is the preferred method for deriving process-related loading vectors when classes are narrowly separated. The effectiveness of the decomposition strategy is demonstrated using artificial spectra and applied to a ToF-SIMS materials science case study on the photodegradation of N719 dye, a common dye in photovoltaics, on a mesoporous TiO_2_ anode. The study revealed that the photodegradation process varies over time, and the resulting fragments have been identified accordingly. The proposed methodology, applicable to both labeled (supervised) and unlabeled (unsupervised) spectral data, can be seamlessly integrated into most modern mass spectrometry data analysis workflows to automatically generate a list of peaks whose relative behavior varies between two processes, and is particularly effective in identifying subtle differences between highly similar physicochemical processes.

## Introduction

The complexity of mass spectrometry (MS) spectra has driven the development of innovative approaches and algorithms to extract valuable information. This is particularly true for hard-ionization methods that achieve mass resolutions high enough to distinguish peaks at the same nominal mass, such as time-of-flight secondary ion mass spectrometry (ToF-SIMS), where fragmentation and numerous isotopic peaks result in complex but highly correlated spectra. Typically, these correlations are captured by high-dimensional peak lists containing the intensities of hundreds of peaks. As a result, multivariate analysis (MVA) techniques, such as principal component analysis (PCA), are routinely applied for dimensionality reduction, aiming to capture these correlations in interpretable, compact forms.^1−5^ This has been demonstrated in various fields, including biology,^4,6,7^ medical sciences,^8−10^ polymer sciences,^11,12^ and organic coatings,^13,14^ with most studies focusing on the analysis of MS images. Concurrently, significant efforts have been made to optimize MVA workflows and implementations specifically for analyzing MS data.^15−24^

The conventional application of PCA in the context of MS typically reveals characteristic differences between spectra; however, it is often crucial to compare the manner in which spectra evolve from one state to another. For instance, two processes may induce different alterations in an initial spectrum, and the objective is to identify the differences in these transformations. More precisely, the goal is to identify peaks whose relative behavior (e.g., increasing or decreasing), compared with all other peaks, differs between the two processes. When the processes are similar, the identification can be time-consuming and prone to error if done traditionally as it necessitates an expert to meticulously inspect the spectra for meaningful differences. In this context, similar processes are those that affect peak intensities in analogous ways, causing the same peaks to increase or decrease in intensity with exceptions primarily related to relative peak intensity differences. Essentially, the majority of spectral changes are consistent for the two processes, with differences evident only in the relative behavior of a few peaks. These scenarios frequently occur when comparing closely related physicochemical processes, such as two nearly identical polymers exposed to the same reagent or treatment, the adsorption of proteins onto multiple similar surfaces, or identical solar cells exposed to slightly different environments. Notably, time-dependent processes can be considered similar when sampled at different times, for example, when comparing samples exposed to an environment or treatment for varying durations. We propose a straightforward strategy that can be integrated into most modern MS data analysis workflows to automatically generate a list of peaks that capture the meaningful differences between two processes. The strategy involves calculating the orthogonal component via Gram-Schmidt decomposition^25^ of two loading vectors obtained by MVA (e.g., PCA), where the loading vectors represent the directions of chemical change for the two processes, and inspecting the peaks with the largest coefficients in this orthogonal component.

While the application of PCA to ToF-SIMS data has become widespread, MS experiments often associate labels to spectra by design, allowing for the use of supervised alternatives. For instance, when it is known that an analyzed region has been exposed to a treatment while another region has not, or to a different extent, or when a spectrum can be associated with a class through information obtained by another method (e.g., imaging), labels are available, and supervised algorithms can be employed. One such example is partial least-squares discriminant analysis (PLS-DA), a straightforward supervised alternative to PCA that also serves as a dimensionality reduction technique by producing scores and loadings.^26,27^ While PCA combines peaks to identify directions of maximal variance, PLS-DA uses labels to find directions of maximal covariance.^28^ More often than not, this direction captures the relevant information and functions as a meaningful discriminator.^3,5,29^ Although the applications of PCA and PLS-DA differ due to PCA’s unsupervised nature, we demonstrate that PLS-DA more reliably captures the direction of change between two classes of spectra when the classes are narrowly separated. However, when the spectral differences between classes are substantial, both methods generally offer equally effective tools for further analysis by using the decomposition strategy.

**Figure 1:**
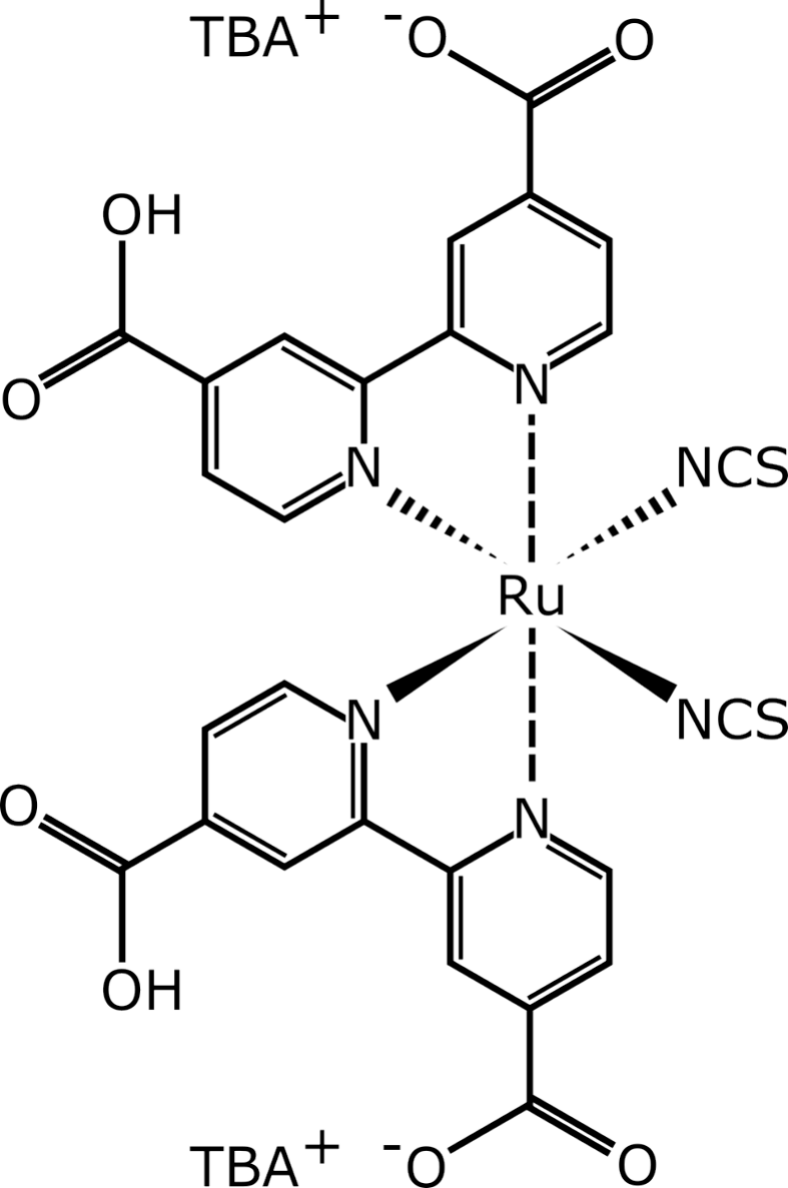
Chemical structure of N719 dye.

The underlying principles of the proposed decomposition strategy are demonstrated using artificial spectra and subsequently applied to a ToF-SIMS materials science case study involving the photodegradation of N719 dye, a commercially available and widely used dye renowned for its efficiency and stability, primarily used in the field of photovoltaics.^30^ Specifically, the dye is commonly employed as a light-absorbing component in dye-sensitized solar cells, where the dye molecules anchor to mesoporous TiO_2_.^31^ The photovoltaic activity of the N719 molecule is linked to its chemical structure, which is shown in Figure 1.^30^ In this case study, the proposed strategy is applied to investigate the photodegradation process of N719 dye on TiO_2_; however, the strategy can, in principle, be applied to any two physicochemical processes.

## Experimental Section

### Sample Preparation

Two samples were prepared, in accordance with the procedure from Hustings et al.,^32^ using presintered transparent TiO_2_ anodes and N719 dye, both purchased from Solaronix (Aubonne, Switzerland). The anodes consist of a mesoporous layer of anatase TiO_2_ nanoparticles (Ti-Nanoxide T/SP) screen-printed onto fluorine-doped tin oxide-coated glass substrates. The anodes were refired at 450 °C for 30 min to remove pollutants from the TiO_2_ layer. After cooling to 60 °C, both anodes were submerged upside down in a 0.3 mmol L^–1^ solution of N719 dye in 96% eurodenatured ethanol, ensuring uniform staining. After overnight staining, the samples were rinsed with the same solvent and air-dried before being attached onto a glass support. Both samples were subsequently partially covered by a metal plate before being placed 30 cm away from the opening of a Xe-arc lamp set at 150 W. After approximately 4 h, the first sample was removed from the support and stored in a dark environment. The second sample was removed the next day for a total exposure of 22 h. Partial covering and exposure to the Xe-arc lamp led to the creation of two distinct regions on the samples. On each sample, the region shielded from light by the metal plate will be referred to as “unexposed,” while the other region will be referred to as “illuminated.” The illuminated regions are distinguished from the unexposed ones by a clear visual discoloration of the dye. A summary of these samples and regions is given in Table 1. Finally, both samples were placed into a custom-made chamber which was flushed with N_2_ for 10 min before being sealed for transport to the ToF-SIMS equipment.

**Table 1:** Summary of the Experimental Features of the Case Study of TiO_2_/N719 Photodegradation and the Associated Spectra Analyzed in This Work

Class	Sample ID	Exposure time (h)	# Spectra	Polarity
Unexposed #1	#1	0	3	–
Unexposed #2	#2	0	3	–
Short-duration illuminated	#1	4	3	–
Long-duration illuminated	#2	22	3	–

### ToF-SIMS Analysis

ToF-SIMS analysis was performed using the TOF.SIMS 5 equipment from IONTOF GmBH (Münster, Germany) located at Imec (Leuven, Belgium). The 30 keV Bi_3_^+^ primary ion beam transmitted a current of approximately 0.2 pA at a cycle time of 200 μs in the so-called high-current bunched mode, which maximizes the mass resolution. Both the unexposed and the illuminated regions of the two N719 stained TiO_2_ samples were each analyzed in three areas of 100 × 100 μm^2^ by randomly scanning over 64 × 64 pixels (per area), collecting negatively charged secondary ions (see Table 1). Additionally, a pure TiO_2_ sample without N719 dye was analyzed to simplify peak identification during the data analysis. Each measurement was terminated after reaching a primary ion dose density of 3 × 10^12^ ions/cm^2^, which is below the static limit of 10^13^ ions/cm^2^. Charge compensation was achieved using a low-energy electron flood gun set at 21 V and a fixed surface potential of −7.7 V. All measurements were performed under a vacuum of 3–8 × 10^–9^ mbar.

### Data Analysis

The ToF-SIMS analysis resulted in a collection of 12 measurements, summarized in Table 1. For each measurement, three files in proprietary file formats were obtained (with extensions .itm, .itmx, and .itax). These files were examined using the proprietary software *SurfaceLab 7.3* from IONTOF GmbH (Münster, Germany), and subsequently calibrated with CH_2_^–^, CH_3_^–^, C_3_H^–^, C_4_H^–^, and C_5_H^–^ (except for the spectra of the long-duration illuminated regions where the C_5_H^–^ peak was too small to be used for calibration). In this study, we analyzed the spectra obtained by averaging over all pixels and all scans, meaning that neither imaging data nor depth profiles were considered. Within *SurfaceLab 7.3*, an automatic peak detection routine was run on one spectrum of each measurement class: unexposed #1, unexposed #2, short-duration illuminated (*t*_*S*_ = 4 h), and long-duration illuminated (*t*_*L*_ = 22 h) . The routine scanned the entire mass range (1–3661 *m*/*z*) for peaks that met the following criteria: a minimum of 100 counts, a signal-to-noise ratio of at least 1.0, a maximum background level of 0.8, and selection based on an adaptive peak width filter. The union of the four automatically generated peak lists was formed by merging peaks within a catch mass radius of 200 ppm, which was determined through trial-and-error to avoid duplicates. Peaks with center masses differing by less than this mass deviation were considered to be equivalent. This final combined peak list was then used as the peak list for all spectra, producing a data set in which all measurements had the same 993 peaks as features, with each peak characterized by the summed number of counts (intensity) between the peak margins at an associated center mass (*m*/*z*). The Poisson dead-time-corrected peak information was exported (as .txt files in UTF-8 encoding) for further analysis using the *Python* programming language.

The data underwent the following preprocessing steps before any MVA: removal of saturated peaks (in this case, only CN^–^ at 26 *m*/*z*), Poisson scaling, and mean centering. The spectra were not normalized by total counts, although this normalization was tested and found to have minimal impact on the results. PCA was performed using the PCA function of the *scikit-learn* library, with all components retained, no whitening, and the “full” option, ensuring that the exact full singular value decomposition was calculated using the standard *LAPACK* solver.^33,34^ Additionally, PLS-DA was conducted with the *scikit-learn* library’s PLSRegression function, using the same number of components, no additional scaling, with up to 500 iterations, and a convergence tolerance of 10^–6^, while employing categorical response variables. The loadings were always back-transformed to account for the prior scaling.^15,16^

### Generation of Artificial Data Sets

Artificial spectra enabling controlled variation in class separation were generated to support the analyses. These artificial spectra were derived by modifying the peak intensities of spectra from the class unexposed #1 using the following procedure. First, half of the peaks in the peak list were randomly selected for modification. For each selected peak *p*_*i*_, the direction of the intensity change (increase or decrease) was determined with equal probability. An update rate *r*_*i*_ was then sampled from a normal distribution  with mean μ and standard deviation σ. For peaks with increasing intensities, an additional update factor *F*_*i*_ was sampled from a uniform distribution . The sampled values were applied according to the following update rules:
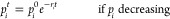
1
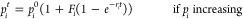
2
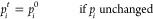
3where *p*_*i*_^0^ denotes the initial peak intensity of the *i*th peak and *p*_*i*_^*t*^ represents the peak intensity after artificial process time *t*. Although *t* is referred to as a time-like parameter for intuitive understanding, it is actually dimensionless and merely serves to indicate the extent of spectral change; therefore, it could be interpreted as another physical or chemical variable that causes spectral changes. For artificial data sets involving a single mechanism, the update rates and factors were sampled as  and , respectively. In the case of two-mechanism artificial data sets, peak intensities were updated twice: first with  and , followed by  and . Finally, the mean spectrum was calculated from the three artificial spectra, and Poisson noise was applied to it three separate times to eliminate any one-to-one correlations with the original spectra. This procedure results in a data set comprising two classes of spectra: three “initial spectra” and three “artificial spectra,” where the degree of class separation is determined by the parameter *t*.

## Results and Discussion

### Comparing PLS-DA and PCA Loading Vectors versus the Target

We first demonstrate that PLS-DA more reliably identifies the loading vector that correctly differentiates between two classes of spectra compared to PCA, particularly when the differences between the classes are minimal. To facilitate this analysis, artificial spectra with controlled variations in class separation, governed by a time-like parameter *t*, were generated. The behavior of the loading vectors obtained by PCA (*L*_PCA_(*t*)) and PLS-DA (*L*_PLSDA_(*t*)) at various times *t* is evaluated by creating 1000 artificial data sets, each based on a distinct, randomly generated artificial process, and subsequently performing PCA and PLS-DA on each generated data set. For each MVA method, the loading vector that maximizes the between-within ratio of the corresponding scores is selected from all components; in the case of PLS-DA, this was consistently the first loading vector. This selection is a crucial step of the comparison procedure, as it simulates an analyst’s decision in choosing which component best discriminates between the two classes. For each data set, the similarity between the selected loading vectors is quantified using the cosine similarity *S*_*C*_, defined for two vectors *V*_1_ and *V*_2_ as
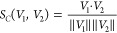
4This similarity measure ranges from −1 for anticorrelated (opposite) vectors to 1 for correlated (parallel) vectors, with 0 indicating uncorrelated (orthogonal) vectors. The signs of loading vectors are manually adjusted so that *S*_*C*_ ∈ [0,1], capturing the same information regardless. The overall behavior across the 1000 data sets is assessed by the mean cosine similarity , which is subsequently transformed into an angle . This angle represents the similarity of the high-dimensional PCA and PLS-DA loading vectors in just two dimensions.

**Figure 2:**
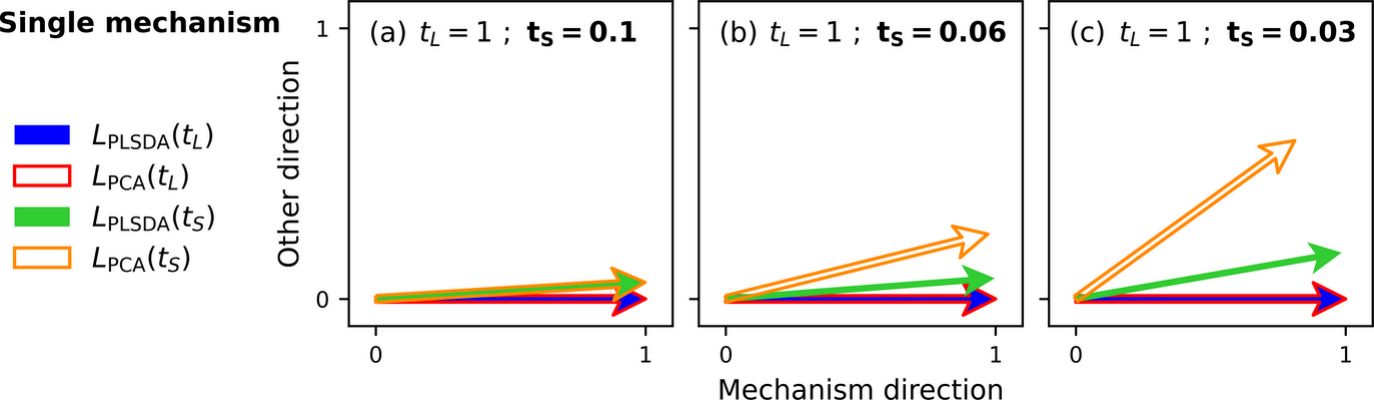
Two-dimensional representations of the loading vectors obtained by PCA and PLS-DA for single-mechanism artificial data sets. The *x*-axis is aligned with the PLS-DA loading vector of a data set in which the original spectra underwent the artificial process for *t*_*L*_ = 1 arbitrary units, yielding well-separated classes along the direction of the mechanism. The PCA loading vector overlaps for *t*_*L*_ = 1, but when the contrast between spectra is decreased by reducing the process time to (a) *t*_*S*_ = 0.1, (b) *t*_*S*_ = 0.06, or (c) *t*_*S*_ = 0.03, then the PLS-DA loading vector captures the mechanism direction more accurately than the PCA loading vector.

The results of this procedure for various artificial process times are depicted in Figure 2. For a long process time *t*_*L*_ = 1, the PCA and PLS-DA loading vectors exhibit nearly complete overlap, indicating that both methods identify the same direction and capture identical information; namely, how peaks contribute to variance (PCA) or class covariance (PLS-DA) in the data sets. This alignment occurs because the difference between initial and artificial spectra after process time *t*_*L*_ = 1 is substantial, aligning the direction of maximum variance with the direction of maximum covariance between the peaks and class labels. Termed “the mechanism direction” in this context, this identified direction reflects the peak intensity changes resulting from the underlying mechanism of the artificial process. For each data set, the mechanism is defined by the randomly sampled values that govern the peak intensity changes (from the initial to the artificial spectra) after a specific process time. Since there is only one set of sampled values, each artificial process has only one underlying mechanism, making the mechanism direction the target. In Figure 2, this direction also serves as a reference, with all other loading vectors being evaluated relative to it by calculating the angle θ, as previously explained. It is important to interpret the two-dimensional representations in Figure 2 judiciously: only the angles between the reference direction (which in this case is *L*_PLSDA_(*t*_*L*_)) and other loading vectors accurately reflect their similarity, as any other pair of vectors may not lie in the same plane.

When the process time is decreased to *t*_*S*_ = 0.1 (Figure 2a), there is only a slight misalignment between the PCA and PLS-DA loading vectors. Further reducing the process time to *t*_*S*_ = 0.06 or *t*_*S*_ = 0.03 (Figure 2b–c) results in greater misalignment, but importantly, the PLS-DA loading vector aligns more closely with the mechanism direction than the PCA loading vector. This signifies that when the differences between spectra in different classes are minimal, PLS-DA more reliably captures the target information compared to PCA, making PLS-DA more effective in low-contrast situations. This is attributable to PLS-DA’s use of class information, which aids in identifying the discriminating direction, whereas PCA’s focus on directions of maximum variance can hinder identification of the target loading vector, especially when class-related differences are small.

### An MVA Strategy to Identify Differences between Similar Processes

The underlying principles of the proposed strategy to identify differences between similar processes are demonstrated by introducing a second mechanism in the artificial processes. In this scenario, the initial peak intensities are first updated according to a fast-changing mechanism, mechanism #1, and subsequently, the same peaks are updated according to a slow-changing mechanism, mechanism #2. This approach mimics a simple time-dependent process affecting spectra differently based on the process time *t*. For instance, spectra generated after a short process time are expected to reflect only mechanism #1 (fast), whereas after a long process time both mechanisms are combined. Consequently, spectra at different process times can be interpreted as originating from different but highly similar processes (instead of from a single time-dependent process) as they incorporate the same two mechanisms but to varying extents.

**Figure 3:**
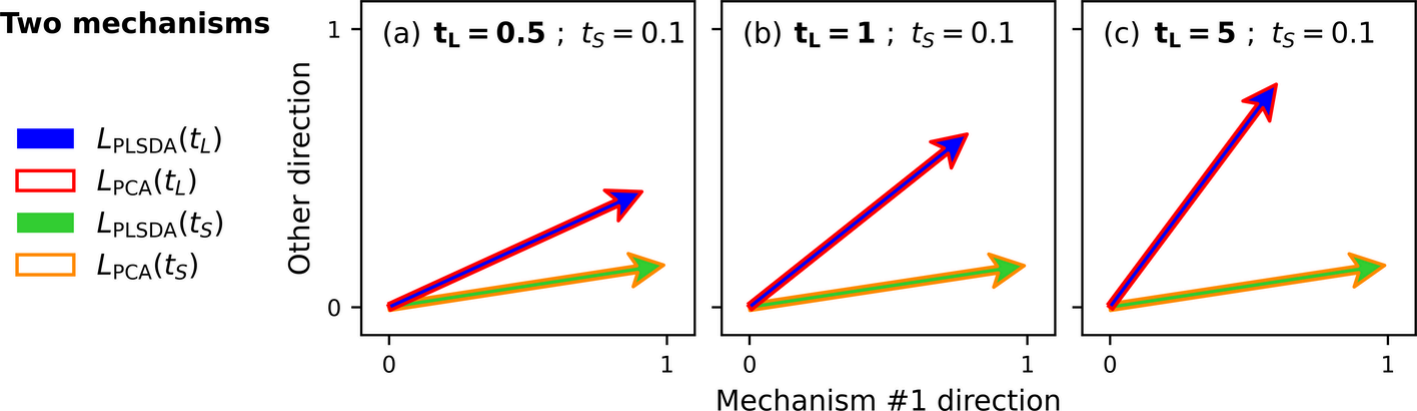
Two-dimensional representations of the loading vectors obtained by PCA and PLS-DA for two-mechanism artificial data sets. The *x*-axis is aligned with the PLS-DA loading vector of a data set in which the original spectra underwent exclusively the first mechanism for *t* = 1 arbitrary units. The PCA and PLS-DA loading vectors overlap in all cases, reflecting that the classes are always well separated, but the loading vectors become increasingly distinct for longer process times, *t*_*S*_ = 0.1 and (a) *t*_*L*_ = 0.5, (b) *t*_*L*_ = 1, and (c) *t*_*L*_ = 5, indicating that the second mechanism is increasingly incorporated at longer process times.

For the two-mechanism situation, loading vectors at various process times are calculated and compared by a procedure similar to that outlined previously for a single mechanism. However, in this case, the loading vectors obtained after short process times *t*_*S*_ and long process times *t*_*L*_ are compared to the loading vector obtained by exclusively acting with mechanism #1 for *t* = 1, referred to as the “mechanism #1 direction.” In Figure 3, the loading vectors of the two-mechanism artificial processes are compared for increasingly longer process times *t*_*L*_. Simultaneously, it is exemplified that the loading vectors after a short process time *t*_*S*_ = 0.1 align significantly with the mechanism #1 direction, confirming that the spectral changes after short process times reflect mainly the fast-changing mechanism. Note that the PCA and PLS-DA loading vectors always overlap, but the discrepancy between the loading vectors and the mechanism #1 direction becomes larger with increasing process times *t*_*L*_. This suggests that the loading vectors in all cases capture the spectral differences attributed to the artificial processes. Notably, the discrepancies are not due to noise signals, which previously was the case for low-contrast single-mechanism artificial processes after short process times *t*_*S*_. Indeed, contrary to the low-contrast single-mechanism case, the loading vectors now all lie approximately in the same plane, as signified by the relations between the angles of different loading vectors. Namely, a typical two-mechanism data set yields an angle between the reference and the long-time loading vector θ(*L*_ref_ → *L*(*t*_*L*_)) and an angle between the reference and the short-time loading vector θ(*L*_ref_ → *L*(*t*_*S*_)) such that their difference is almost equal to the angle between the short-time loading vector and the long-time loading vector θ(*L*(*t*_*S*_) → *L*(*t*_*L*_)) (e.g., one of the data sets at *t*_*L*_ = 1 had θ(*L*_ref_ → L(*t*_*L*_)) – θ(*L*_ref_ → L(*t*_*S*_)) = 32.72° ≈ 33.68° = θ(*L*(*t*_*S*_) → *L*(*t*_*L*_))). These observations indicate that for increasing process times, the loading vectors become more distinct from mechanism #1 due to a systematic incorporation of mechanism #2. This, in turn, implies that the *y*-axis in Figure 3 is highly correlated to what we would call the “mechanism #2 direction,” or more precisely, to the difference between the two mechanisms, which will now be demonstrated more explicitly.

**Figure 4:**
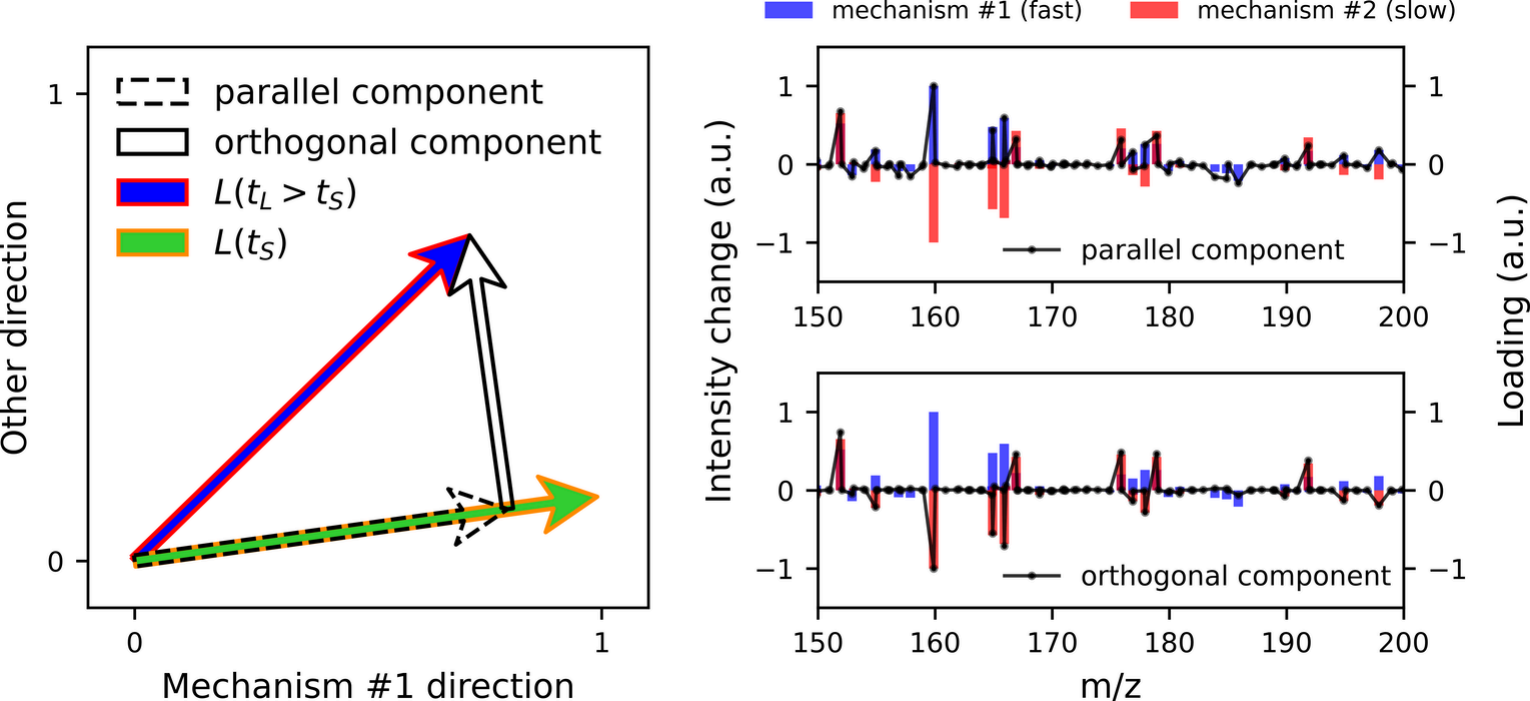
Left: Two-dimensional representation of the loading vectors obtained by PCA and/or PLS-DA for two-mechanism artificial data sets. The *x*-axis is aligned with the PLS-DA loading vector of a data set in which the original spectra underwent exclusively the first mechanism for *t* = 1 arbitrary units. The loading vectors *L*(*t*_*S*_) and *L*(*t*_*L*_ > *t*_*S*_) capture the directions of spectral change of an artificial time-dependent process at *t*_*S*_ = 0.1 and *t*_*L*_ = 2, respectively, while the decomposition of *L*(*t*_*L*_ > *t*_*S*_) into its parallel and orthogonal components is shown. Right: An artificial data set is generated by modifying the peak intensities of initial spectra according to two mechanisms, thereby producing the second class of spectra. The average intensity changes in the data set after process time *t*_*L*_ due to mechanism #1 (blue bars) and mechanism #2 (red bars) are strongly correlated with the parallel component (top figure) and the orthogonal component (bottom figure), respectively. The *m*/*z* range is chosen arbitrarily; this behavior is generally observed across the entire *m*/*z* range. This strategy thus enables the decomposition of two processes into a shared component (∼mechanism #1) and a component that maximally differentiates the two processes (∼mechanism #2).

The core idea of the strategy to identify differences between similar processes is that the loading vectors associated with these processes span a plane in which the loading vectors can be decomposed in a meaningful way. Perhaps the most straightforward decomposition of two vectors is given by the Gram-Schmidt decomposition into the parallel component *P* and the orthogonal component *O* of a vector *V*_1_ projected onto a vector *V*_2_ as^25^parallel component:
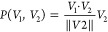
5orthogonal component:
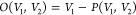
6For our particular case of loading vectors, *L*(*t*_*L*_) is projected onto *L*(*t*_*S*_), as illustrated in Figure 4 (left). When the short process time *t*_*S*_ is sufficiently small and mechanism #1 (#2) is sufficiently fast (slow), then *L*(*t*_*S*_) adequately approximates the mechanism #1 direction. As illustrated in Figure 4 (right), the artificial processes have the benefit that the two mechanisms making up the virtual single time-dependent process can be inspected separately. Concretely, the intensity changes from each mechanism are individually accessible because they are calculated before they are added to the initial spectra. Comparing the intensity changes from each mechanism to the parallel and orthogonal components, as presented in Figure 4 (right), with blue bars for mechanism #1, and red bars for mechanism #2, reveals a strong correlation between the parallel component and mechanism #1, and between the orthogonal component and mechanism #2. Essentially, the long-time loading vector *L*(*t*_*L*_ > *t*_*S*_) contains significant contributions from both mechanisms (fast and slow), reflecting that it is obtained from a data set that contains initial spectra and spectra after a long-time process, whereas the short-time loading vector *L*(*t*_*S*_) contains almost exclusively contributions from mechanism #1 (fast). Therefore, the orthogonal component can be seen to “filter out” mechanism #1 (out of *L*(*t*_*L*_ > *t*_*S*_)), leaving only that which maximally differentiates the two processes, which in this case corresponds approximately to mechanism #2 (slow), as also depicted in Figure S1. This exemplifies how this decomposition strategy allows a single time-dependent process to be separated into a short-time scale part and a long-time scale part. More generally, without any reference to time, this strategy decomposes two similar processes into: 1) a component that the two processes have in common and 2) a component that maximally differentiates the two processes.

### Case Study: Photodegradation of N719 Dye on Mesoporous TiO_2_

**Figure 5:**
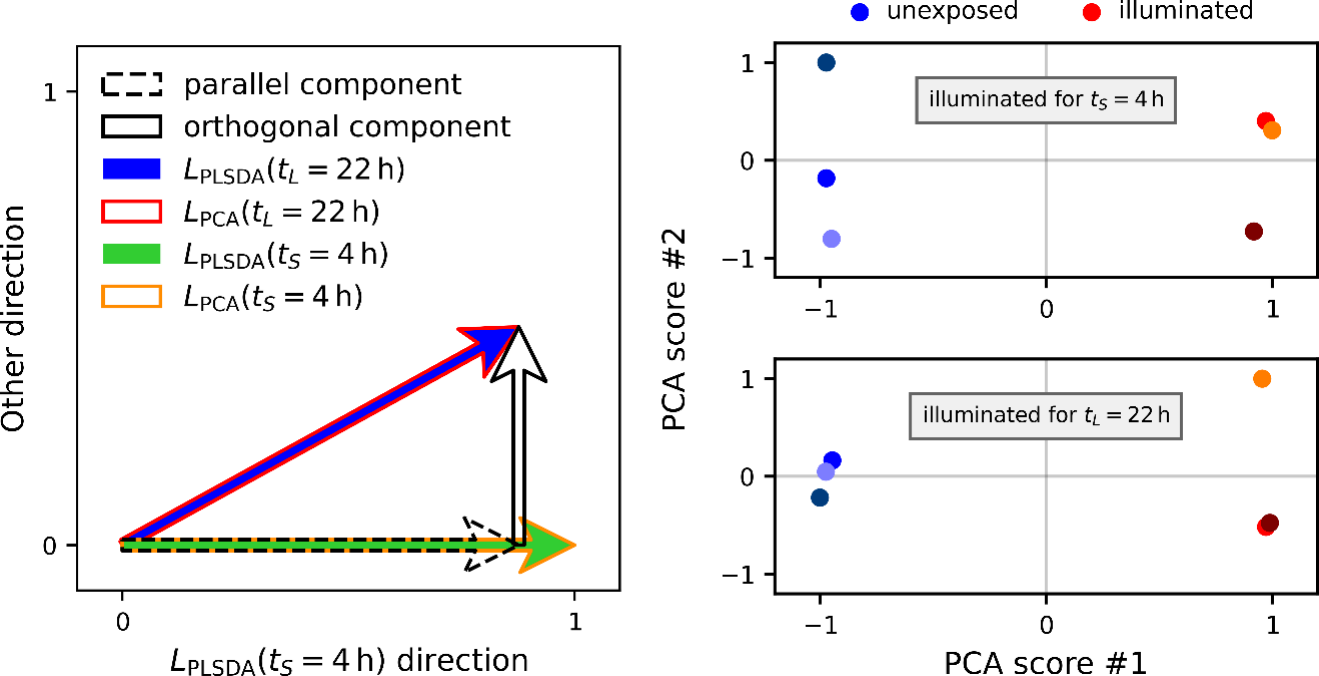
Left: Two-dimensional representation of the loading vectors obtained by PCA and PLS-DA for the two experimental N719 data sets. Two samples were half photodegraded by exposure to light for different durations *t*_*S*_ = 4 h and *t*_*L*_ = 22 h, while the other half of each sample remained unexposed, which defined the two classes of the two data sets. The PCA and PLS-DA loading vectors overlap; however, the loading vectors for different illumination durations point in distinct directions, indicating the time-dependent nature of the photodegradation process. Right: For both *t*_*S*_ = 4 h and *t*_*L*_ = 22 h, the first loading vector captures the difference between the unexposed and illuminated spectra, as evidenced by the class separation along the *x*-axis in the scores plot.

Two N719-stained TiO_2_ samples were selectively photodegraded by exposure to light for varying durations. The first sample resulted in a data set of spectra classified as unexposed #1 and short-duration illuminated (*t*_*S*_ = 4 h), while the second sample led to a data set of spectra classified as unexposed #2 and long-duration illuminated (*t*_*L*_ = 22 h). The left side of Figure 5 shows that the PCA and PLS-DA loading vectors associated with the data sets for both illumination durations overlapped, signifying that, in this specific case study, both methods are equally effective at identifying the discriminating directions of the processes. However, the loading vectors for different illumination durations were significantly distinct. Importantly, there was clear class separation in both cases, evidenced by the PCA scores on the right side of Figure 5 (the PLS-DA scores showed similar class separation, not shown). These observations suggest that the photodegradation process is time-dependent, indicating that the direction of chemical change varies with the illumination duration. Thus, the processes associated with the two illumination durations can be considered similar, and the developed decomposition strategy can be used to identify meaningful differences between them.

**Figure 6:**
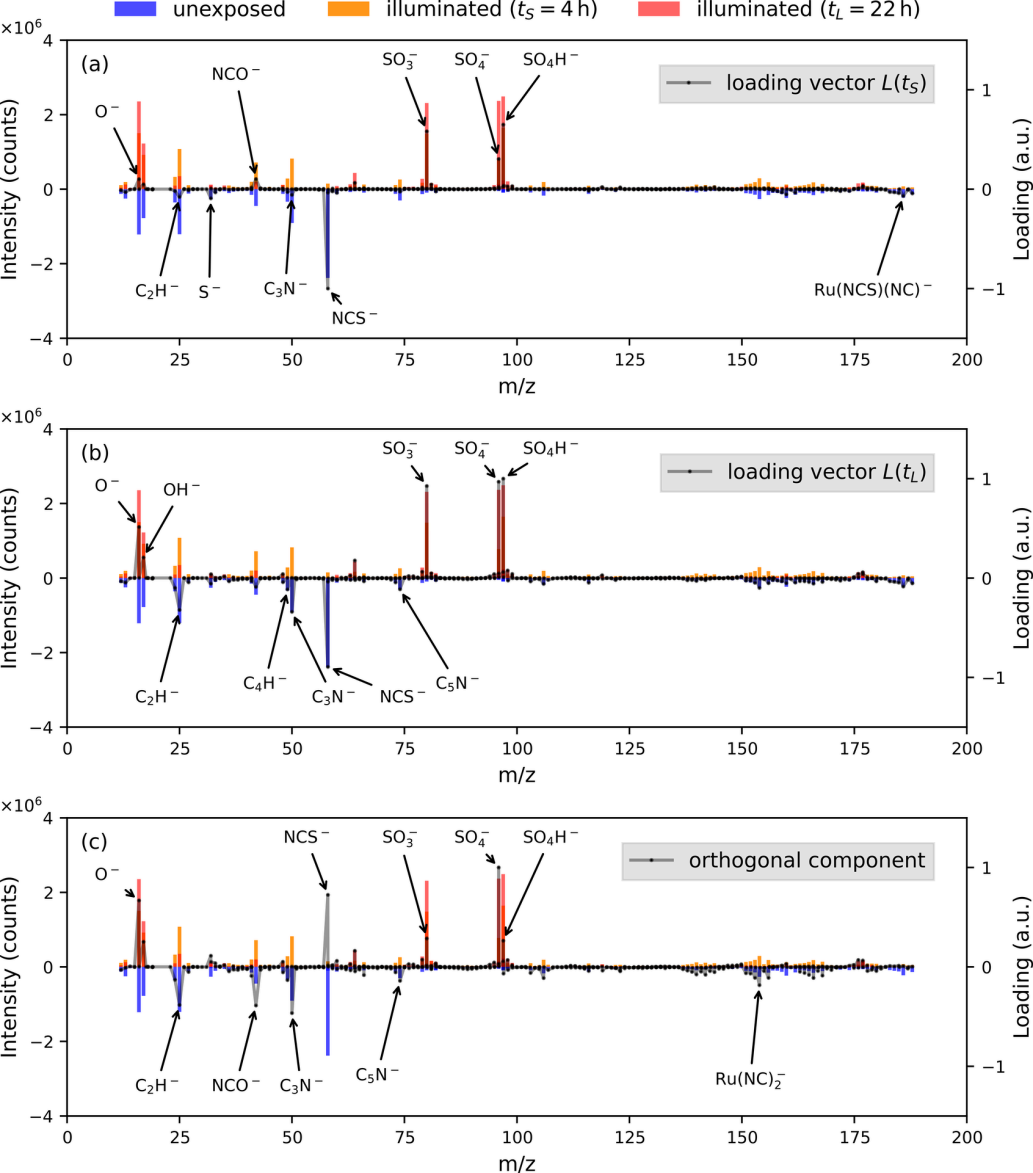
Average peak intensities of unexposed (blue bars), short-duration illuminated (*t*_*S*_ = 4 h) (orange bars) and long-duration illuminated (*t*_*L*_ = 22 h) (red bars) spectra versus the PLS-DA loadings (dotted lines). The comparison is shown for (a) the loading vector *L*(*t*_*S*_) obtained by comparing unexposed to short-duration illuminated spectra, (b) the loading vector *L*(*t*_*L*_) obtained by comparing unexposed to long-duration illuminated spectra, and (c) the orthogonal component. To enhance clarity, average peak intensities for unexposed spectra are plotted on the negative *y*-axis. Peaks with the five largest positive and negative coefficients are annotated in each case. It is evident that the orthogonal component incorporates information from both loading vectors *L*(*t*_*S*_) and *L*(*t*_*L*_).

The average spectra for each measurement class are presented in Figure 6 (note that spectra for unexposed #1 and unexposed #2 are combined due to their near-identical nature, as shown later). This figure includes the short-duration illuminated loading vector (Figure 6a), the long-duration illuminated loading vector (Figure 6b), and the orthogonal component (Figure 6c). Peaks associated with the five largest positive and negative coefficients for each loading vector are annotated. This annotation reveals that the loading vectors *L*(*t*_*S*_) and *L*(*t*_*L*_) share several common peaks, such as O^–^, SO_3_^–^, SO_4_^–^, C_2_H^–^, C_3_N^–^, and NCS^–^, suggesting that the photodegradation involves the photooxidation of thiocyanate and the production of sulfur oxides. However, differences between the vectors are also evident, as seen in peaks such as OH^–^, NCO^–^, S^–^, C_4_H^–^, Ru(NCS)(NC)^−^, and C_5_N^–^, which indicate that the involvement of certain peaks in the photodegradation process varies with the illumination duration, underscoring the time-dependent nature of the photodegradation process. Remarkably, the orthogonal component highlights unique peaks (e.g., NCO^–^), as well as peaks shared by *L*(*t*_*S*_) and *L*(*t*_*L*_) (e.g., SO_4_^–^), and also identifies additional new peaks (e.g., Ru(NC)_2_^–^).

**Figure 7:**
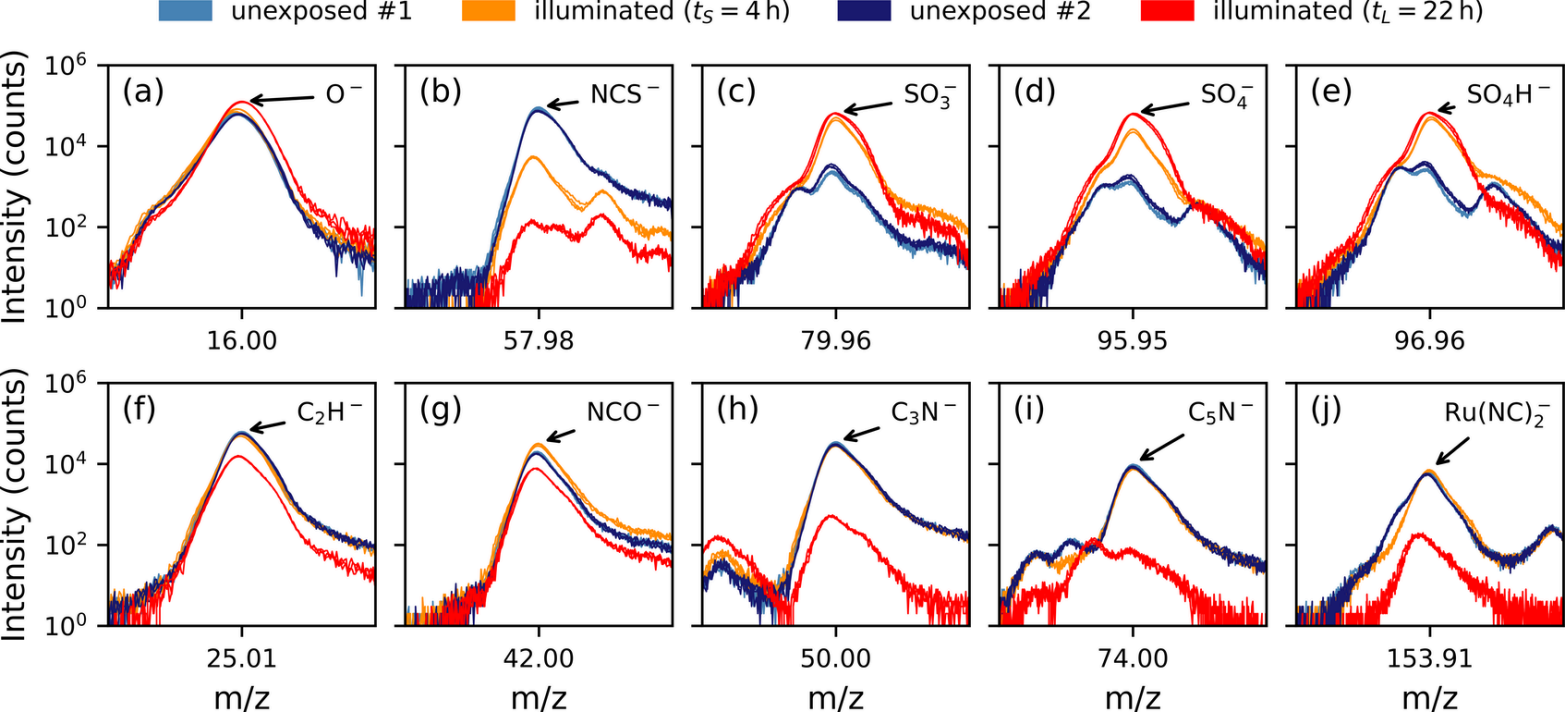
Spectral intensities of unexposed, short-duration illuminated (*t*_*S*_ = 4 h), and long-duration illuminated (*t*_*L*_ = 22 h) regions of N719-stained TiO_2_. Spectra classified as “unexposed #1” are from the same sample (but a different region) as those classified as “illuminated (*t*_*S*_ = 4 h),” and similarly for “unexposed #2” and “illuminated (*t*_*L*_ = 22 h).” The *m*/*z* windows are centered around the peaks with the five largest positive (a-e) and negative (f-j) coefficients of the orthogonal component defined in the main text.

To elucidate the incorporation of certain peaks into the orthogonal component, a closer examination of the peaks with the five largest positive and negative coefficients is presented in Figure 7. It is evident that all of these peaks exhibit significant intensity changes upon illumination, with varying degrees depending on the illumination duration. Strikingly, after illuminating for 4 h, the intensities of NCO^–^ and Ru(NC)_2_^–^ increase, whereas after 22 h, these intensities decrease (Figure 7g,j). This suggests that initial illumination may remove a sulfur atom from the thiocyanate, leading to an increase in Ru(NC)_2_^–^, potentially followed by an oxygen atom replacement that initially increases NCO^–^. However, on a longer time scale, these molecules also degrade, resulting in decreased intensities of Ru(NC)_2_^–^ and NCO^–^. Conversely, the peaks of C_2_H^–^, C_3_N^–^, and C_5_N^–^ remain nearly constant after illuminating for 4 h, but decrease substantially after 22 h (Figure 7f,h,i). The pronounced dependence of these peaks’ intensities on illumination duration indicates that molecular structural changes suppress the detection of the corresponding fragments, an effect occurring only after prolonged illumination. Potential reasons include the slow generation of certain reactive species or the disappearance of highly reactive components, such as thiocyanate, which may shield other molecular parts by reacting swiftly with reactive species. This hypothesis is supported by the complete disappearance of thiocyanate (Figure 7b) and the apparent stagnation of sulfur oxide production (Figure 7c–e). While prior studies have identified thiocyanate ligands as primary degradative components in N719 photodegradation,^35,36^ the direct evidence of sulfur oxide production via ToF-SIMS and the time-dependent nature of this photodegradation process, as reported here, contribute new insights and will be incorporated into a broader study on the underlying mechanism of light-induced patterning of N719-stained photoanodes for “photovoltaic photographs.”^a^

## Conclusions

A strategy was developed to discern differences between similar processes, such as variations in relative spectral changes between comparable treatments or exposures causing related physicochemical transformations. For instance, in time-dependent ToF-SIMS analyses, where samples are evaluated after different durations, the varying exposure times can be interpreted as multiple similar processes. The strategy involves decomposing a loading vector (representing one process) into its parallel and orthogonal components when projected onto another loading vector (representing a different process). The orthogonal component reveals peaks whose relative behavior—such as the direction and magnitude of intensity changes—varies between the two processes, thereby identifying significant differences between them. In this context, it was demonstrated that when two classes of spectra are narrowly differentiated, then PLS-DA more reliably captures the loading vector that discriminates between the classes, compared to PCA. This improved performance of PLS-DA is due its use of class information, which reduces the impact of noise signals and other sources of variance and enhances the identification of the discriminating direction. However, when the classes are well separated, both PLS-DA and PCA typically identify the same direction of interest, providing equally effective tools for further analysis.

The decomposition strategy was applied to a ToF-SIMS case study of N719 dye photodegradation on mesoporous TiO_2_. Two loading vectors were calculated using PCA and PLS-DA: one discriminating unexposed from short-duration illuminated spectra and another discriminating unexposed from long-duration illuminated spectra. The analysis indicated that the photodegradation process is time-dependent. Decomposing the loading vectors resulted in an orthogonal component that highlighted peaks strongly associated with long-time scale photodegradation characteristics and those that vary disproportionately across the probed time scales. This exemplifies how the decomposition strategy can effectively identify differences among similar processes by leveraging spectral data.

The practical applications of this strategy are far reaching as it enhances routinely used MVA techniques and workflows. While demonstrated here in the context of time-dependent processes, it is broadly applicable to any two similar physicochemical processes. This strategy is especially useful for ToF-SIMS and mass spectrometry techniques using other ionization methods, such as matrix-assisted laser desorption/ionization or desorption electrospray ionization,^10,37−39^ with potential applications in biology, forensics, organic chemistry, energy, and medical sciences.

## Supporting Information Available

Figure of loading vector decomposition demonstrated with artificial data sets, showing the average intensity changes associated with both mechanisms and their correlation with (decomposed) loading vectors of the artificial processes (PDF)

## Footnotes

a Robert, A et al. On the Underlying Mechanism of Light-Induced Patterning of N719-Stained Photoanodes for “Photovoltaic Photographs”. **2024**, Manuscript in preparation
