# Supplementary material for: Identifying Process Differences with ToF-SIMS: An MVA Decomposition Strategy
Source: J Am Soc Mass Spectrom. 2024 Oct 4;35(12):3116–25. doi: 10.1021/jasms.4c00327 (PMC11622371; doi:10.1021/jasms.4c00327)
Supplement: Supplementary file 1 — js4c00327_si_001.pdf [file js4c00327_si_001.pdf]

Supporting Information for Manuscript: "Identifying Process Differences with ToF-SIMS: An MVA Decomposition Strategy"

Nico Fransaert,[*,†] Allyson Robert,[†] Bart Cleuren,[‡] Jean V. Manca,[*,†] and Dirk Valkenborg[*,¶]

†*UHasselt, X-LAB, Agoralaan, 3590 Diepenbeek, Belgium*

‡*UHasselt, Theory Lab, Agoralaan, 3590 Diepenbeek, Belgium*

¶*UHasselt, Data Science Institute, Interuniversity Institute for Biostatistics and Statistical Bioinformatics, Center for Statistics, Agoralaan, 3590 Diepenbeek, Belgium*

E-mail: nico.fransaert@uhasselt.be; jean.manca@uhasselt.be; dirk.valkenborg@uhasselt.be

Loading Vector Decomposition Demonstrated with Artificial Data Sets

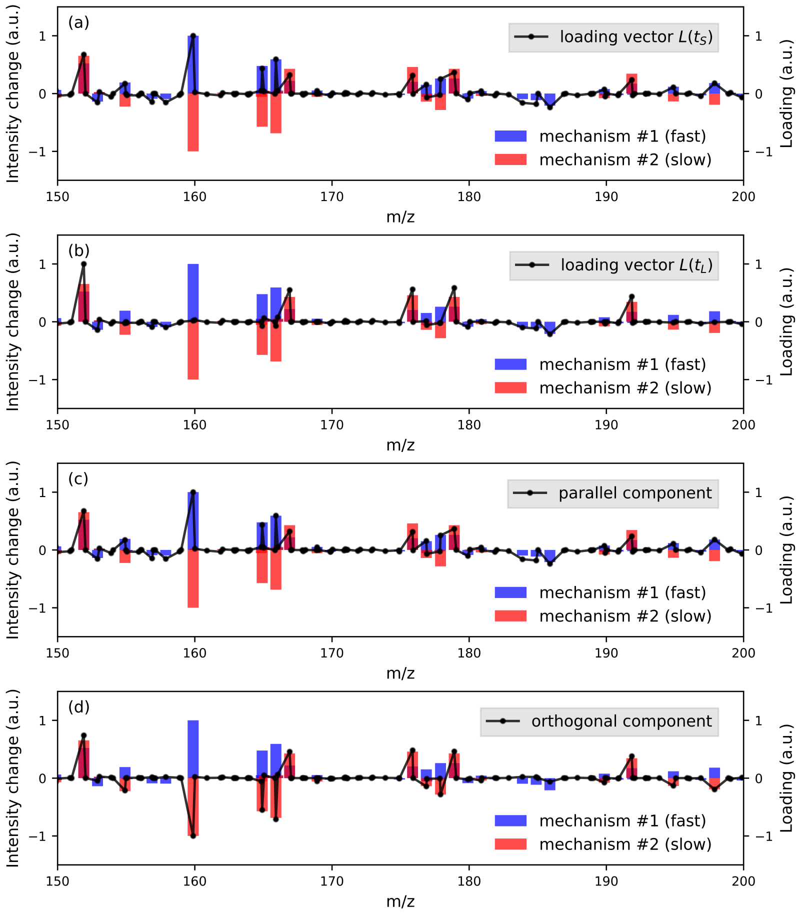

Figure S1: An artificial data set is generated by modifying the peak intensities of initial spectra according to two mechanisms, thereby producing the second class of spectra. The average intensity changes in the data set after process time $t_L = 2$ due to mechanism #1 (blue bars) and mechanism #2 (red bars) are compared to (a) the loading vector after a short process time $L(t_S = 0.1)$, (b) the loading vector after a long process time $L(t_L = 2)$, (c) the parallel component, and (d) the orthogonal component, obtained as discussed in the main text. The loading vectors incorporate both mechanisms but to different extents, due to the mechanisms' distinct time dependencies. The parallel component is equivalent to $L(t_S)$ by construction. The orthogonal component "filters out" the contribution of mechanism #1 (fast) in $L(t_L)$, isolating purely mechanism #2 (slow), thereby facilitating the clear identification of mechanism #2. Lines connecting the dots of the loadings are included to guide the eye. The m/z range is chosen arbitrarily; this behavior is generally observed across the entire m/z range. This strategy thus enables the decomposition of two processes into a shared component (∼mechanism #1) and a component that maximally differentiates the two processes (∼mechanism #2).
